# Feasibility of a checklist in treating hypertension in primary care – base line results from a cluster-randomised controlled trial (check and support)

**DOI:** 10.1186/s12872-018-0963-5

**Published:** 2018-12-19

**Authors:** Aapo Tahkola, Päivi Korhonen, Hannu Kautiainen, Teemu Niiranen, Pekka Mäntyselkä

**Affiliations:** 1 Institute of Public Health and Clinical Nutrition, University of Eastern Finland, Kuopio, Finland; 2 Health Centre of Jyväskylä Cooperation Area, Jyväskylä, Finland; 3 University of Turku, Turku, Finland; 4 Medcare Oy, Äänekoski, Finland; 5 National Institute for Health and Welfare, Helsinki, Finland; 6 Division of Medicine, Turku University Hospital, Turku, Finland; 7 Department of Medicine, University of Turku, Turku, Finland; 8 Primary Health Care Unit, Kuopio University Hospital, Kuopio, Finland

**Keywords:** Hypertension, Initiation, Medication, Target, Checklist, IMB model, Primary care

## Abstract

**Background:**

Most patients with antihypertensive medication do not achieve their blood pressure (BP) target. The most important factor behind this failure is poor medication adherence. However, non-adherence to therapy does not concern only patients. Clinicians also tend to lack adherence to hypertension guidelines, overestimate BP control and be satisfied with inadequate BP control. The aim of this non-blinded, cluster-randomised, controlled study was to investigate if using a checklist would improve the quality of care in the initiation of new antihypertensive medication and help reduce non-adherence.

**Methods:**

The study was conducted in eight primary care study centres in Central Finland, randomised to function as either intervention (*n* = 4) or control sites (n = 4). We included patients aged 30–75 years who were prescribed antihypertensive medication for the first time. Initiation of medication in the intervention group was carried out with a 9-item checklist, filled in together by the treating physician and the patient. Hypertension treatment in the control group was managed by the treating physician without a study-specific protocol.

**Results:**

In total, 119 patients were included in the study, of which 118 were included in the analysis (*n* = 59 in the control group, n = 59 in the intervention group). When initiating antihypertensive medication, an adequate BP target was set for 19% of the patients in the control group and for 68% in the intervention group. Shortly after the appointment, only 14% of the patients in the control group were able to remember the adequate BP target, compared with 32% in the intervention group. The use of the checklist was also related to more regular agreement on the next follow-up appointment (64% in the control group versus 95% in the intervention group). No adverse events or side effects were related to the intervention.

**Conclusions:**

Even highly motivated new hypertensive patients in Finnish primary care have significant gaps in their informational and behavioural skills. The use of a checklist for initiation of antihypertensive medication was related to significant improvement in these skills. Based on our findings, the use of a checklist might be a practical tool for addressing this problem.

**Trial registration:**

NCT02377960. Date of registration: February 26th, 2015.

## Background

Most patients on antihypertensive medication do not achieve their blood pressure (BP) target in Finland [1], Europe [2] or worldwide [3]. This results in a vast amount of preventable cardiovascular complications, especially in patients with other cardiovascular risk factors or prevalent heart disease [4, 5] BP control rates remain poor year after year, even though some clear improvements have also been reported, for example from England [6]. The most important factor behind this failure is poor medication adherence [7]. During the first year of treatment, patients have been shown to possess antihypertensive medication only 50% of the time [8]. However, non-adherence to therapy does not concern only patients. Clinicians also tend to lack adherence to hypertension guidelines, overestimate BP control and be satisfied with inadequate BP control [9–11].

Several other barriers to successful hypertension treatment are also well known, such as health care providers’ disagreement with clinical recommendations and patients’ lack of knowledge, stress, anxiety or depression [12]. However, very little attention has been focused on patients’ knowledge of their target BP. From 9 to 51% of hypertensive patients do not know their BP target, even though having this knowledge is associated with improved BP control [13–15]. Novel ways of addressing barriers to successful hypertension treatment are therefore clearly needed. One option for improving the quality of patient care seems to be implementation of a checklist [16]. Checklists are decision aids traditionally more commonly used in non-medical industries. Some evidence suggests that checklists could also improve the quality of patient care in medical settings [16] and have a positive effect on treatment compliance [17]. To our knowledge, however, there is no evidence of the use of checklists in the initiation of antihypertensive medication in outpatient care.

The aim of this cluster-randomised, controlled study was to investigate if using a checklist would improve the quality of care in the initiation of new antihypertensive medication and help reduce non-adherence. In this report we focus on the baseline characteristics of the study patients and the effect of a checklist on patient knowledge.

## Methods

The Check and Support Study (ClinicalTrials.gov reference NCT02377960) was a cluster-randomised controlled study carried out in a primary care setting. The aim of the study was to assess whether a checklist for initiation of antihypertensive medication, combined with personalised Short Messaging Service (SMS) text message support, would result in improved systolic BP control as compared with usual care during the initial 12 months of therapy. The study was conducted according to the principles of the Declaration of Helsinki and it adheres to CONSORT (Consolidated Standards of Reporting Trials) 2010 guidelines.

### Setting

The study was conducted in eight primary care study centres in Central Finland. The study centres included five public sector health centres, one private occupational care centre and one public sector health centre that also provided occupational health care. Together, these centres provide primary care services for a population of approximately 200,000 persons. All the study centres were first grouped into comparable pairs. The pairs were set to match the following essential features: location (urban-rural), centre size and selection of services (occupational health care service or not). The study centres were then randomised to the intervention (*n* = 4) or control (*n* = 4) arms (two-cluster design) by one of the authors (AT), using a two-block randomisation list. All the centres in both arms received the basic study information and a short lesson on current hypertension treatment guidelines, including setting an adequate BP target.

### Patients

Patients were recruited by treating physicians when initiating a new antihypertensive medication in routine practice between January 27th 2015 and March 6th, 2018, until sufficient amount of patients were recruited. We included patients aged 30–75 years who were (1) starting antihypertensive medication for the first time with (2) a clinical diagnosis of hypertension, (3) a mobile phone, (4) ability to read text messages, (5) ability to take care of their personal medication, (6) ability to perform home BP measurements (7) and an agreement for using electric drug prescriptions (standard care in Finland).

**Fig. 1:**
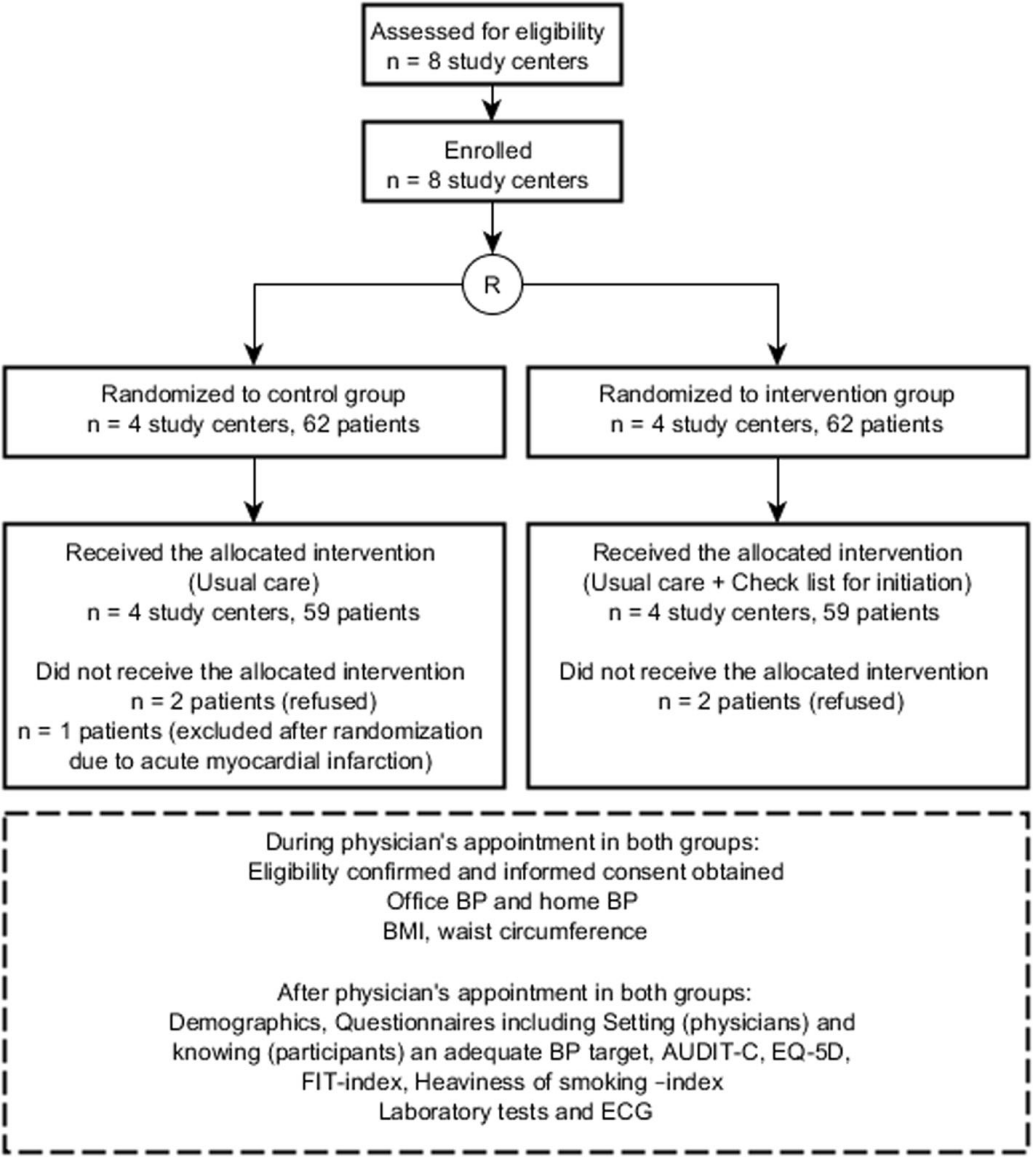
Flow of the study. AUDIT-C: alcohol use disorders identification test; BMI: Body Mass Index; BP: Blood pressure; ECG: electrocardiogram; EQ-5D: EuroQoL questionnaire of health-related quality of life; FIT index: Frequency-Intensity-Time (FIT) Index, Laboratory tests: fasting plasma glucose level, fasting plasma cholesterol level, existence of proteinuria, creatinine level

Exclusion criteria were: (1) having or suspected of having depression or psychosis, (2) a malignant disease that was determined to have an impact on life expectancy, (3) atrial flutter or atrial fibrillation, (4) pregnancy, (5) unwillingness to give informed consent and take part in the study, (6) systolic BP > 200 mmHg, (7) diastolic BP > 120 mmHg, (8) rapid onset or worsening of hypertension, (9) kidney disease, defined as an estimated glomerular filtration rate < 45 ml/min/1.73m^2^, hypokalemia (K < 3.3 mmol/l) or proteinuria (albumin-creatinine ratio > 30 mg/mmol, night urine albumin > 200 μg/min, 24-h protein excretion > 500 mg/day, or urine dipstick test showing proteinuria). The study flow is presented in Fig. 1.

### Baseline measurements

Each patient’s baseline office BP, height, weight and waist circumference were measured by the treating physician. Office BP was measured three times after sitting still for at least 5 min, from the left arm with a Microlife WatchBP Home A or N automatic oscillometric monitor [18]. A wide-range (arm circumference 22–42 cm) semi-rigid conical cuff was used as a default, but large (arm circumference > 42 cm) and small cuffs (arm circumference < 22 cm) were available.

Immediately after the initial appointment, all the patients filled in a questionnaire on basic demographics, smoking habits (Heaviness of Smoking Index) [19] and alcohol use with alcohol consumption questions (AUDIT-C) from the alcohol use disorders identification test (AUDIT) [20]. We used a preference-based, five-dimensional instrument (EQ-5D) to measure health-related quality of life, with the value 1 indicating best possible health [21]. To assess exercise habits, we used the Frequency-Intensity-Time (FIT) Index (Kasari D.: Effects of exercise and fitness on serum lipids in college women, Unpublished). The score range is 1–100; points < 36 indicate low, 37–63 moderate and 64 or more high physical activity.

An electrocardiogram was taken and the following lab tests were done for blood samples obtained after the initial appointment: fasting plasma cholesterol and fasting plasma glucose (photometric, enzymatic method, measured after at least 8 h of fasting), plasma potassium (ion selective electrode, indirect method), plasma creatinine (photometric, enzymatic method) and estimated glomerulus filtration rate (eGFR, the Chronic Kidney Disease Epidemiology Collaboration CKD-EPI equation) [22]. Proteinuria was measured with the albumin excretion rate measured from nightly urine (cU-Alb, immunoturbidimetry .method), diurnal urinary protein excretion (dU-Prot, turbidometry method) or spot urine albumin-creatinine ratio (U –AlbKre, U -Alb: immunoturbidimetry method/U -Krea: photometric, enzymatic method).

Office blood pressure was defined as the mean of three measurements. University- or college-level education was considered higher education. Body Mass Index (BMI) was calculated by dividing the patient’s weight (kg) by the square of his/her height (m).

Systematic COronary Risk Evaluation system (SCORE) was used to calculate ten year cardiovascular risk for study participants [23]. The SCORE system estimates the ten year risk of a first fatal atherosclerotic event in relation to age, sex, systolic blood pressure, smoking and total cholesterol level. Risk level is then categorized to low (< 1%), moderate (≥1 to < 5%), high (5–10%) or very high risk (≥10%). However, patients with documented CVD, diabetes, very high levels of individual risk factors, or chronic kidney disease (stages 3–5) are automatically considered to be at very high or high ten year CV risk, without formal CV risk estimation. The presence of hypertension-mediated organ damage can also increase CV risk to a higher level.

### Intervention

The Information-Motivation-Behavioural skills (IMB) model provides both a theoretical basis and a practical guide for designing novel ways to promote medication adherence [24]. We used the IMB model as a theoretical framework to design intervention tools and to detail relevant contents for medication adherence promotion in patients with hypertension. The IMB model suggests that disease-specific information is a crucial prerequisite for adherence, but is not sufficient alone. In addition to information, also motivation and adequate behavioural skills are necessary during treatment. It has been previously demonstrated that concentrating on information alone is not an effective way to motivate the patient [25]. On the other hand, the patient may have good motivation to take medication but treatment can still fail because of inaccurate or insufficient information.

**Fig. 2:**
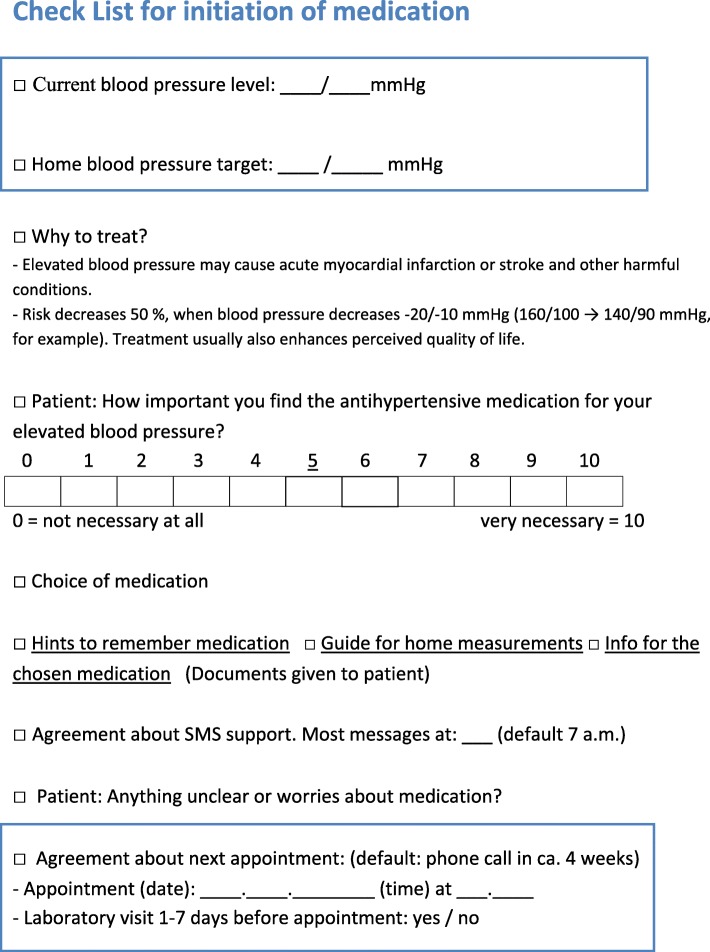
Checklist for initiation of medication. After filling in the checklist with the clinician, the patients received a copy of it for themselves, together with enclosed written information. Underlined sections refer to written information enclosed with the checklist. This information included five alternative medication guides depending on the physician’s choice. IMB model relations: Items 1, 2 and 9 concerned behavioural skills; items 5, 6 (documents given to the patient) and 8 concerned Information; items 3 and 4 concerned motivation and item 7 was an agreement on launching SMS text message support

Initiation of medication in the intervention group was carried out with a checklist consisting of nine items (Fig. 2). The checklist was filled in together by the treating physician and the patient. The checklist included three items on hypertension knowledge, two items on motivation and three items on behavioural skills. One item was an agreement to participate in SMS text message support for 12 months. The checklist was designed to improve the setting of a clear BP target and to guide the conversation to cover the most relevant matters related to medication adherence, such as perceived necessity of medication, and disease-specific behavioural skills, such as agreement on the first follow-up visit. It also sought to assure that the patient was provided with basic information on hypertension and antihypertensive medication. After filling in the checklist, the patients received a copy of it for themselves, together with enclosed written information. Hypertension treatment in the control group was managed by the treating physician without a study-specific protocol.

### Outcomes

Study outcomes were collected with a questionnaire immediately after consultation, before the patients received a copy of the checklist. Thus, the fulfilled checklist was not available when patients answered the query. The outcomes are reported in Table 1, categorised according to the three elements of the IMB model: informational, motivational and behavioural skills (Table 1. Study outcomes).

**Table 1:** Study outcomes

*Outcome*	*Question or other assessment method*	*Scale or interpretation*
*IMB element: Informational*		
Perceived knowledge of home BP target	*“Do you know your personal BP treatment target?”*	Yes or No
Able to report correct home BP target	*“What is your personal BP treatment target?*” (___/___mmHg.)	The home BP target was considered adequate if it was reported to be < 135/85 mmHg (diabetics < 135/75 mmHg).
Home BP target set	Mentioned in the checklist (intervention group) or in electric health record (control group).	Yes, if any written target was found.
Correctly set home BP target	Mentioned in the checklist (intervention group) or in electric health record (control group).	The BP target was considered adequate if it was set at < 135/85 mmHg (< 135/75 mmHg for diabetics).
Perceived understanding of the pharmacodynamics of the medication	“*Do you know how the medication prescribed to you works?”*	Yes or No
Perceived knowledge about potential side effects of the medication	“*Do you know the typical side effects of the antihypertensive medication prescribed to you?”*	Yes or No
Patients reporting to have received written information on the medication	“*Did you receive written information on the antihypertensive medication during the physician’s appointment?”*	Yes or No
Patients reporting to have received written guidance on home BP measurements	*“Did you receive written guidance on how to carry out blood pressure measurements at home*?”	Yes or No
Perceived uncertainty about the medication	*“Did you have any questions about the medication that were left unanswered?”*	Yes or No
*IMB element: Motivational*		
Perceived necessity of antihypertensive medication	*“How necessary do you find the antihypertensive medication for your elevated blood pressure?”*	11-point numerical rating scale (0 = not necessary at all, 10 = very necessary).
Perceived quality of consultation	*“How well did you find that the consultation (including initiation of medication) went in general?”*	11-point numerical rating scale (0 = very bad, 10 = very good).
Perceived degree of difficulty of starting antihypertensive medication	“*How easy or difficult do you find the start and use of antihypertensive medication?”*	11-point numerical rating scale (0 = very difficult, 10 = very easy).
Perceived degree of patient-centeredness	*“Do you think you were able to take part in decision-making sufficiently during the physician’s consultation?”*	Yes or No
IMB element: Behavioural skills		
Agreement on the next appointment	*“Did you arrange the next appointment with your treating physician to evaluate if the medication is suitable and sufficient?”*	Yes or No
Perceived self-confidence for successful treatment	*“Do you believe that the drug treatment of hypertension will succeed?”*	Yes or No
Perceived knowledge about how to act in case of medication side effects	*“Do you know how to act if you get medication side effects?”*	Yes or No
Perceived knowledge about how to act in case that BP target is not reached	*“Do you know how to act if your blood pressure target is not reached?”*	Yes or No

Abbreviations. IMB (model), Information-Motivation-Behavioural skills (model); BP, Blood pressure

The adequate office BP target was considered to be < 140/90 mmHg for most individuals and < 140/80 mmHg for diabetics, in accordance with the then-current European and Finnish Society of Hypertension guidelines [26, 27]. For home BP, the respective targets were < 135/85 mmHg and < 135/75 mmHg. No study-specific medication protocol was used. Only the written notes of the electronic medical record (EMR) and the Check List for initiation of medication were taken into account when assessing if the treating physician had set a blood pressure target.

### Statistical analyses

Continuous variables were analysed using either a t-test or a permutation test, and categorical variables were compared using chi-square or Fisher’s exact test where appropriate. The normality of the variables was tested by using the Shapiro-Wilk W test. The Stata 15.0, StataCorp LP (College Station, TX, USA) statistical package was used for the analysis.

### Sample size calculations

We carried out a power analyses based on the study hypothesis to determine a sufficient amount of patients. We hypothesised that the proportion of patients achieving the systolic BP target in the 12-month follow-up would be 24% in the control group based on studies of Finnish primary care patients, and that the proposed intervention would improve the proportion to 50% [28, 29]. The sample size was estimated using iterative models according to cluster randomisation principles. A sample size of 140 (70 in each group) patients per group was calculated to detect a significant difference with a power of 80% by the two-side α = 0.05. However, due to a slower recruitment rate than expected, we were able to recruit only 119 patients.

## Results

### Baseline characteristics

In total, 119 patients were included in the study, of which 118 were included in the analysis and received intended intervention. One patient was excluded from the study due to acute myocardial infarction before all the baseline measurements were done. Table 2 shows the baseline characteristics of the study patients (Table 2, Patients’ baseline characteristics).

**Table 2:** Patients’ baseline characteristics

Characteristics	Intervention	Control	*P*-value
Total	59	59	
Female, n (%)	39 (66)	35 (59)	0.45
Mean age, years (SD)	58 (11)	58 (10)	0.89
Higher education, n (%)	21 (36)	9 (15)	0.011
Married or co-habiting, n (%)	45 (76)	47 (80)	0.66
Working, n (%)	31 (53)	26 (44)	0.41
Diabetes mellitus, n (%)	5 (9)	7 (12)	0.54
Lifestyle and quality of life			
FIT index (Physical activity) (SD)	40 (19)	36 (20)	0.35
AUDIT-C index (Alcohol use) (SD)	3.3 (2.7)	3.3 (2.5)	0.97
Heaviness of smoking index (SD)	9 (15)	11 (19)	0.62
Health-related quality of life index (EQ-5D) (SD)	0.86 (0.18)	0.86 (0.16)	0.91
Office systolic BP, mmHg (SD)	172 (20)	173 (20)	0.87
Office diastolic blood pressure, mmHg (SD)	101 (12)	102 (13)	0.72
Home systolic BP, mmHg (SD)	156 (15)	152 (13)	0.20
Home diastolic blood pressure, mmHg (SD)	91 (7)	93 (8)	0.50
Total cholesterol, mmol/l (SD)	5.44 (1.17)	5.46 (1.13)	0.92
LDL cholesterol, mmol/l (SD)	3.19 (1.02)	3.27 (1.10)	0.71
HDL cholesterol, mmol/l (SD)	1.60 (0.47)	1.58 (0.47)	0.83
Triglycerides, mmol/l (SD)	1.37 (1.42)	1.49 (0.70)	0.59
eGFR, ml/min/1.73 m2 (SD)	89 (16)	91 (14)	0.52
Fasting glucose, mmol/l	5.85 (0.91)	6.13 (1.25)	0.19
BMI, kg/m2	28.9 (4.4)	30.5 (6.0)	0.10
10-year SCORE risk			
Low (< 1%), n (%)	13 (23)	10 (17)	
Moderate (≥1 to < 5%), n (%)	33 (60)	37 (63)	
High (5–10%), n (%)	7 (12)	10 (17)	
Very high, (≥10%), n (%)	2 (3)	1 (1)	
Mean risk (SD)	2.53 (2)	2.55 (2)	0.99

Abbreviations: *AUDIT-C* alcohol consumption questions from the alcohol use disorders identification test (AUDIT); *BMI* Body Mass Index; *BP* blood pressure; *eGFR* estimated glomerulus filtration rate (CKD-EPI equation); *EQ-5D* EuroQoL questionnaire of health-related quality of life; *FIT index* Frequency-Intensity-Time (FIT) Index; *HDL* high-density lipoprotein; *LDL* low-density lipoprotein; *SCORE* Systematic COronary Risk Evaluation system

### Outcomes

When initiating antihypertensive medication, the correct BP target was set for 19% of the patients in the control group and for 68% in the intervention group. Shortly after the appointment, the majority of patients in both groups reported knowing their BP target. When asked specifically, however, only 14% of the hypertensive patients in the control group were able to report their correct treatment target. Furthermore, one-third (36%) of the patients in the control group did not know after their appointment when and where the first follow-up visit was supposed to take place.

The patients in the intervention group reported significantly higher informational and behavioural skills. The proportion of patients knowing the correct BP target was 32%, as compared with 14% in the control group. The use of the checklist was also related to more regular agreement on the first follow-up appointment (64% in the control group versus 95% in the intervention group). The patients in the intervention group also reported enhanced understanding of the pharmacodynamics of how the medication worked and increased possession of written information on the medication and home BP measurements. Perceived necessity of antihypertensive medication was significantly high in both groups, with no significant between-group difference.

**Fig. 3:**
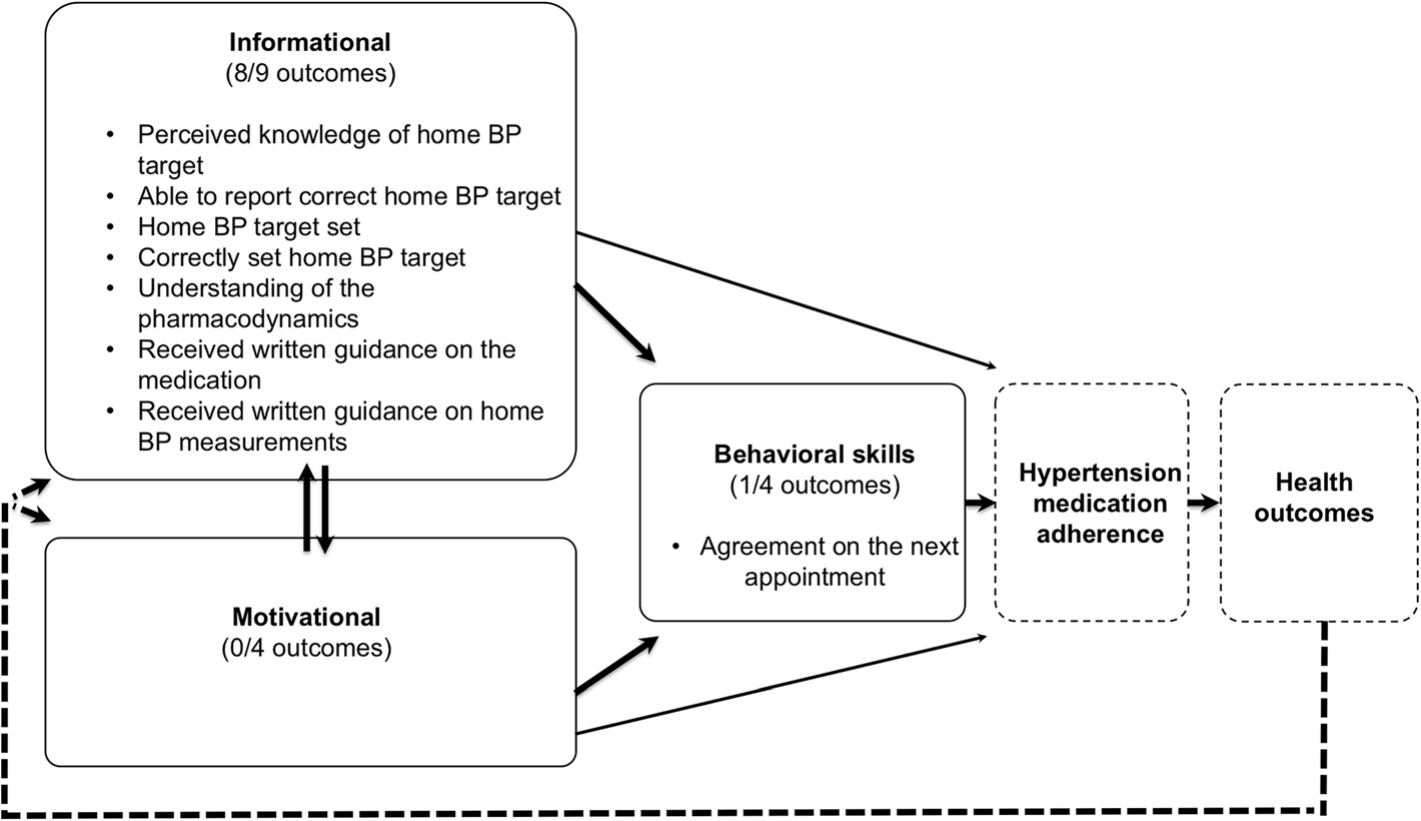
Outcomes in the context of the IMB model. Positive study outcomes in the context of the Information-Motivation-Behavioural skills model for medication adherence. Picture presents only the outcomes that differed significantly between the groups

As a whole, the intervention group had significantly better results in eight of the nine informational outcomes, none of the four motivational outcomes and one of the four behavioural outcomes at the baseline. We found no statistically significant negative effects of intervention. All outcomes are presented in Table 3 and significant differences also in Fig. 3.

**Table 3:** Comparison between study groups according to the IMB model

Outcome	Intervention	Control	P-value
Informational			
*Perceived knowledge of home BP target, n (%), yes*	57 (97)	47 (80)	0.008
*Able to report correct home BP target, n (%), yes*	19 (32)	8 (14)	0.016
*Home BP target set, n (%), yes*	59 (100)	24 (42)	< 0.001
*Correctly set home BP target, n (%), yes*	40 (68)	11 (19)	< 0.001
*Perceived understanding of the pharmacodynamics of the medication, n (%), yes*	49 (83)	37 (63)	0.013
*Perceived knowledge about potential side effects of the medication, n (%), yes*	44 (75)	34 (58)	0.052
*Patients reporting to have received written guidance on the medication, n (%), yes*	53 (90)	23 (39)	< 0.001
*Patients reporting to have received written guidance on home BP measurements, n (%), yes*	54 (92)	40 (68)	< 0.001
*Perceived uncertainty about medication, n (%), yes*	7 (12)	4 (7)	0.34
Motivational			
*Perceived necessity of antihypertensive medication (0 = not necessary at all, 10 = very necessary)*	8.72 (1.32)	9.01 (1.29)	0.26
*Perceived quality of consultation (0 = very bad, 10 = very good)*	9.18 (1.04)	9.17 (1.33)	0.99
*Perceived degree of difficulty to start antihypertensive medication (0 = very difficult, 10 = very easy)*	8.49 (2.14)	8.53 (1.93)	0.97
*Perceived degree of patient-centeredness, n (%), yes*	56 (95)	56 (95)	1.00
Behavioural skills			
*Agreement on the next appointment, n(%), yes*	56 (95)	38 (64)	< 0.001
*Perceived self-confidence for successful treatment, n(%), yes*	57 (97)	59 (100)	0.50
*Perceived knowledge about how to act in case medication side effects occur, n(%), yes*	50 (85)	45 (76)	0.24
*Perceived knowledge about how to act in case the BP target is not reached, n(%), yes*	41 (69)	36 (61)	0.33

Abbreviations: *IMB model* Information-Motivation-Behavioural skills model; *BP* Blood pressure

## Discussion

In this randomised, controlled study we showed that even highly motivated new hypertensive patients in Finnish primary care have significant gaps in informational and behavioural skills and that the use of a checklist for initiation of antihypertensive medication was related to significant improvement in these skills. Especially, only 14% of the hypertensive patients in the control group were able to report the correct treatment target shortly after initiation of the first antihypertensive medication compared with 32% in the intervention group. Furthermore, one-third (36%) of the patients in the control group did not know after the appointment when and where the first follow-up for antihypertensive medication was supposed to take place. Agreement on the next follow-up visit was more regular in the intervention group (95%). We found no statistically significant negative effects of intervention.

### Strengths and limitations

Our study has several strengths. First, while previous studies have usually included individuals that have already received treatment for hypertension, we studied newly diagnosed hypertensive patients in a primary care setting. Randomised trials performed in non-academic settings on newly diagnosed hypertensive patients have been quite scarce thus far. Second, we had a strong theoretical basis (IMB model) when designing a new practical tool for improving the quality of care in the initiation of antihypertensive medication. Third, the study population was representative of a typical general practitioner’s patient population and the study findings are therefore widely applicable to primary care.

Our study also has some limitations. First, the patients were expected to perform home BP measurements for the diagnosis of hypertension. However, several patients in both groups (*n* = 18 in the intervention group, *n* = 17 in the control group) reported having performed the measurements and were able to report their home BP level, but did not bring the complete list of measurements to the clinic. With these patients, the treating physicians decided to trust the patients’ narrative and initiated the medication. However, we were unable to include their home BP measurements in the study analysis. Second, the intervention had the greatest impact on the informational element, some effect on behavioural skills, but very limited effect on the motivational element of the IMB model. We believe this was mainly because behavioural skills and motivational issues were at a high level in the control group. For instance, perceived necessity of antihypertensive medication was graded nine on a scale of one to ten in both groups at the baseline. Therefore, it is not surprising that the intervention had no effect on this outcome. Nevertheless, it is possible that carrying out the intervention during a brief normal physician’s appointment may result in a more hurried patient contact, possibly leading to a decreased feeling of patient centeredness and less time for motivational discussion. Third, contrary to our expectations, perceived uncertainty about medication was higher in the intervention group. Although we did not observe a significant difference between groups, the extra information that the intervention group received might have caused initial confusion in some patients. Even though more information should decrease uncertainty over time, patients did not necessarily take time to read all the information before filling the study questionnaire. Fourth, although the BP target used in our study was based on the then-current European and Finnish Hypertension Guidelines, the uniform target for all study participants is not in full accordance with the most recent European Hypertension guidelines [23]. BP targets introduced in the 2018 ESC guideline are more age-dependent, which would have resulted in different BP targets in different age groups. Finally, this study evaluated only the immediate effects of checklist use. Future studies will determine if the use of a checklist also leads to improvement in medication adherence behaviour and BP control.

### Comparison with existing literature

Some earlier evidence suggests that checklists could improve the quality of patient care in medical settings [16]. Checklists also seem to have a positive effect on treatment compliance, at least in a hospital inpatient care setting [17]. So far, we have had no evidence if that is also true in outpatient care.

Identification of relevant treatment barriers and paying attention to them when designing new interventions makes them more likely to improve practice [30]. Barriers to successful hypertension treatment are quite well studied [12]. All three elements of the IMB model—information, motivation and behavioural skills—are present in them. However, it is somewhat surprising that accurate knowledge of the treatment target is not often mentioned as an important barrier to successful treatment. This is interesting, particularly when earlier studies have demonstrated that patients’ general knowledge of their individual BP target is quite poor, at least in patients with established CHD [14]. Furthermore, patients’ knowledge about the BP target has been associated with improved hypertension control in multiple patient cohorts [13–15, 31].

Multiple IMB model-based interventions in different patient groups have been proven effective in enhancing medication adherence [32–34], but there seem to be no earlier studies of IBM model-based interventions in the initiation of antihypertensive medication or adherence to BP medication.

### Implications for research and practice

This study offers a good starting point for further investigation of the IMB model and the use of a checklist in the initiation of therapy in different patient groups. We still need a better understanding of what the most essential elements of an effective checklist are. One potential focus for a future study could be to investigate the use of a checklist in less motivated patients, and with a wide enough timeframe to truly enable motivational discussion, if needed. Furthermore, the validity of the IMB model in improving the quality of care and medication adherence among primary care hypertensive patients needs to be examined further. We also call for more studies investigating BP target setting in real life primary care settings. Although certain interventions have been demonstrated to work in academic settings, these findings may not apply in primary care [35].

As for implications for practice, this study is a strong reminder for practicing physicians that a clear treatment target and an unambiguous treatment plan should be identified as necessary elements of successful treatment. It is indefensible that one-third of the patients in the control group did not know when and where the first follow-up visit after initiation of medication was supposed to take place. In addition, only a small minority in the control group knew the adequate BP target shortly after initiation of antihypertensive medication. When we assessed whether the treating physician had set the blood pressure target correctly, we only took into account only written notes that could be found from the EMR or from the Check List for initiation of medication. In addition, some clinicians might have communicated the treatment target to the patient only verbally. However, we consider that a verbally agreed treatment target alone is not sufficient for the good care. Furthermore, despite the up-to-date educational sessions for clinicians at the beginning of the study, some clinicians in both groups set the treatment target wrong. Typically the target was set higher than what is recommended in the current guidelines.

Implementing a checklist for the initiation of antihypertensive medication might improve patient care quality in daily practice. In our study, the use of a checklist was related to increased occurrence of setting an adequate BP target, the patient knowing the correct BP target, and agreeing on a follow-up visit. However, some patients even in the intervention group had an inadequate treatment target with no clear clinical reason for it. We assume that the effectiveness of a checklist might still increase if it included a default BP target (e.g. < 135/85 mmHg) so that a physician would not have to remember the current guidelines, but only accept or change the default target when discussing it with the patient.

This study also makes it very clear how counter-intuitive and hard-to-remember a BP target is for a patient. We believe that a BP follow-up system with a personalised, simultaneous graphical demonstration of the patient’s current BP level and BP target level would take us still one step closer to successful BP treatment. This approach would reduce the need to remember numbers by heart and would offer more intuitive feedback for both patients and clinicians. Furthermore, it is important to notice that the checklist does not necessarily need to be a physical list on paper. “Checklist thinking” might as well be applied to a digital self-care portal for hypertensive patients.

Based on our findings, a checklist for initiation of antihypertensive medication might be a practical tool for primary care physicians with which to improve the quality of care. However, before having follow-up results we do not know if the use of a checklist leads to better treatment adherence and BP control in the long term. Future studies also need to confirm our findings in different patient groups. Furthermore, it has to be considered that patients’ adherence to anti-hypertensive care not only depends on the amount of time spent with practitioner or the quality of consultation. Adherence also depends on also on the side effects of the chosen drug and its promptness in relieving symptoms.

## Conclusions

This cluster-randomised, controlled study showed that even highly motivated new hypertensive patients in Finnish primary care often have significant gaps in their disease-specific informational and behavioural skills. The use of a checklist, filled in together by the treating physician and the patient when initiating new antihypertensive medication, was related to significant improvement in these skills. The most important differences between the groups were better knowledge of the correct treatment target (14% in the control group, 32% in the intervention group) and more regular agreement on the next follow-up visit (64% in the control group, 95% in the intervention group).

Based on this study, a checklist for initiation of antihypertensive medication seems to be a practical tool for primary care physicians with which to improve the quality of care. However, we do not recommend implementation of checklists before having follow-up results and before future studies have confirmed our findings in different patient groups.

## Abbreviations

AUDIT: Alcohol use disorders identification test
AUDIT-C: Alcohol consumption questions from AUDIT
BMI: Body Mass Index
BP: Blood pressure
ECG: Electrocardiogram
eGFR: Estimated glomerulus filtration rate (CKD-EPI equation)
EMR: Electric medical record
EQ-5D: EuroQoL questionnaire on health-related quality of life
FIT index: Frequency-Intensity-Time (FIT) Index
HDL: High-density lipoprotein
IMB model: Information-Motivation-Behavioural skills model
LDL: Low-density lipoprotein
SCORE: Systematic COronary Risk Evaluation system
SMS: Short Messaging Service text message

## References

[1] Kastarinen M, Antikainen R, Peltonen M, Laatikainen T, Barengo NC, Jula A, Salomaa V, Jousilahti P, Nissinen A, Vartiainen E, Tuomilehto J (2009). Prevalence, awareness and treatment of hypertension in Finland during 1982-2007. J Hypertens.

[2] Kotseva K, De Bacquer D, De Backer G, Ryden L, Jennings C, Gyberg V, Abreu A, Aguiar C, Conde AC, Davletov K, Dilic M, Dolzhenko M, Gaita D, Georgiev B, Gotcheva N, Lalic N, Laucevicius A, Lovic D, Mancas S, Milicic D, Oganov R, Pajak A, Pogosova N, Reiner Z, Vulic D, Wood D, On Behalf Of The Euroaspire Investigators (2016). Lifestyle and risk factor management in people at high risk of cardiovascular disease. A report from the European Society of Cardiology European Action on Secondary and Primary Prevention by Intervention to Reduce Events (EUROASPIRE) IV cross-sectional survey in 14 European regions. Eur J Prev Cardiol..

[3] Chow CK, Teo KK, Rangarajan S, Islam S, Gupta R, Avezum A, Bahonar A, Chifamba J, Dagenais G, Diaz R, Kazmi K, Lanas F, Wei L, Lopez-Jaramillo P, Fanghong L, Ismail NH, Puoane T, Rosengren A, Szuba A, Temizhan A, Wielgosz A, Yusuf R, Yusufali A, McKee M, Liu L, Mony P, Yusuf S (2013). PURE (Prospective Urban Rural Epidemiology) Study investigators. Prevalence, awareness, treatment, and control of hypertension in rural and urban communities in high-, middle-, and low-income countries. JAMA.

[4] Waeber B, Feihl F (2013). Assessment of drug compliance in patients with high blood pressure resistant to antihypertensive therapy. EuroIntervention.

[5] Iannaccone M, Quadri G, Taha S, D’Ascenzo F, Montefusco A, Omede P, Jang IK, Niccoli G, Souteyrand G, Yundai C, Toutouzas K, Benedetto S, Barbero U, Annone U, Lonni E, Imori Y, Biondi-Zoccai G, Templin C, Moretti C, Luscher TF, Gaita F (2016). Prevalence and predictors of culprit plaque rupture at OCT in patients with coronary artery disease: a meta-analysis. Eur Heart J Cardiovasc Imaging.

[6] Falaschetti E, Mindell J, Knott C, Poulter N (2014). Hypertension management in England: a serial cross-sectional study from 1994 to 2011. Lancet.

[7] Sabate E (2003). Adherence to long-term therapies: Evidence for action. 1st ed.

[8] Gwadry-Sridhar FH, Manias E, Lal L, Salas M, Hughes DA, Ratzki-Leewing A, Grubisic M (2013). Impact of interventions on medication adherence and blood pressure control in patients with essential hypertension: a systematic review by the ISPOR medication adherence and persistence special interest group. Value Health.

[9] Bramlage P, Thoenes M, Kirch W, Lenfant C (2007). Clinical practice and recent recommendations in hypertension management--reporting a gap in a global survey of 1259 primary care physicians in 17 countries. Curr Med Res Opin.

[10] Redon J, Erdine S, Bohm M, Ferri C, Kolloch R, Kreutz R, Laurent S, Persu A, Schmieder RE (2011). SHARE steering committee. Physician attitudes to blood pressure control: findings from the supporting hypertension awareness and research Europe-wide survey. J Hypertens.

[11] Inkster M, Montgomery A, Donnan P, MacDonald T, Sullivan F, Fahey T (2005). Organisational factors in relation to control of blood pressure: an observational study. Br J Gen Pract.

[12] Khatib R, Schwalm JD, Yusuf S, Haynes RB, McKee M, Khan M, Nieuwlaat R (2014). Patient and healthcare provider barriers to hypertension awareness, treatment and follow up: a systematic review and meta-analysis of qualitative and quantitative studies. PLoS One.

[13] Majernick TG, Zacker C, Madden NA, Belletti DA, Arcona S (2004). Correlates of hypertension control in a primary care setting. Am J Hypertens.

[14] Prugger C, Keil U, Wellmann J, de Bacquer D, de Backer G, Ambrosio GB, Reiner Z, Gaita D, Wood D, Kotseva K, Heidrich J, EUROASPIRE III (2011). Study group. Blood pressure control and knowledge of target blood pressure in coronary patients across Europe: results from the EUROASPIRE III survey. J Hypertens.

[15] Wright-Nunes JA, Luther JM, Ikizler TA, Cavanaugh KL (2012). Patient knowledge of blood pressure target is associated with improved blood pressure control in chronic kidney disease. Patient Educ Couns.

[16] Hales B, Terblanche M, Fowler R, Sibbald W (2008). Development of medical checklists for improved quality of patient care. Int J Qual Health Care.

[17] Wolff AM, Taylor SA, McCabe JF (2004). Using checklists and reminders in clinical pathways to improve hospital inpatient care. Med J Aust.

[18] Stergiou GS, Giovas PP, Gkinos CP, Patouras JD (2007). Validation of the microlife WatchBP home device for self home blood pressure measurement according to the international protocol. Blood Press Monit.

[19] Heatherton TF, Kozlowski LT, Frecker RC, Rickert W, Robinson J (1989). Measuring the heaviness of smoking: using self-reported time to the first cigarette of the day and number of cigarettes smoked per day. Br J Addict.

[20] Bush K, Kivlahan DR, McDonell MB, Fihn SD, Bradley KA (1998). The AUDIT alcohol consumption questions (AUDIT-C): an effective brief screening test for problem drinking. Ambulatory care quality improvement project (ACQUIP). Alcohol use disorders identification test. Arch Intern Med.

[21] Rabin R, de Charro F (2001). EQ-5D: a measure of health status from the EuroQol group. Ann Med.

[22] Levey AS, Stevens LA, Schmid CH, Zhang YL, Castro AF, Feldman HI, Kusek JW, Eggers P, Van Lente F, Greene T, Coresh J (2009). CKD-EPI (chronic kidney disease epidemiology collaboration). A new equation to estimate glomerular filtration rate. Ann Intern Med.

[23] Williams B, Mancia G, Spiering W, Agabiti Rosei E, Azizi M, Burnier M (2018). 2018 ESC/ESH guidelines for the management of arterial hypertension. Eur Heart J.

[24] Fisher JD, Fisher WA, Amico KR, Harman JJ (2006). An information-motivation-behavioral skills model of adherence to antiretroviral therapy. Health Psychol.

[25] Haynes RB, Ackloo E, Sahota N, McDonald HP, Yao X. Interventions for enhancing medication adherence. Cochrane Database Syst Rev 2008; doi: 10.1002/14651858.CD000011.pub3 10.1002/14651858.CD000011.pub3 18425859

[26] ESH/ESC Task Force for the Management of Arterial Hypertension (2013). 2013 practice guidelines for the management of arterial hypertension of the European Society of Hypertension (ESH) and the European Society of Cardiology (ESC). J Hypertens.

[27] Working group appointed by the Finnish Medical Society Duodecim and the Finnish Hypertension Society. Hypertension. Current Care Guideline 2014. http://www.kaypahoito.fi/web/english/guidelineabstracts/guideline?id=ccs00014. Accessed 1 Jun 2018.

[28] Varis J, Savola H, Vesalainen R, Kantola I (2011). Both hypertensive men and women are inadequately treated in Finnish general practice. J Am Soc Hypertens.

[29] Varis J, Savola H, Vesalainen R, Kantola I (2009). Treatment of hypertension in Finnish general practice seems unsatisfactory despite evidence-based guidelines. Blood Press.

[30] Baker R, Camosso-Stefinovic J, Gillies C, Shaw EJ, Cheater F, Flottorp S, Robertson N. Tailored interventions to overcome identified barriers to change: effects on professional practice and health care outcomes. Cochrane Database Syst Rev. 2010. 10.1002/14651858.CD005470.pub2. 10.1002/14651858.CD005470.pub2 PMC4164371 20238340

[31] Knight EL, Bohn RL, Wang PS, Glynn RJ, Mogun H, Avorn J (2001). Predictors of uncontrolled hypertension in ambulatory patients. Hypertension.

[32] Fisher JD, Amico KR, Fisher WA, Cornman DH, Shuper PA, Trayling C, Redding C, Barta W, Lemieux AF, Altice FL, Dieckhaus K, Friedland G (2011). LifeWindows team. Computer-based intervention in HIV clinical care setting improves antiretroviral adherence: the LifeWindows project. AIDS Behav.

[33] Mannheimer SB, Morse E, Matts JP, Andrews L, Child C, Schmetter B, Friedland GH (2006). Terry Beirn community programs for clinical research on AIDS. Sustained benefit from a long-term antiretroviral adherence intervention. Results of a large randomized clinical trial. J Acquir Immune Defic Syndr.

[34] Zarani F, Besharat MA, Sadeghian S, Sarami G (2010). The effectiveness of the information-motivation-behavioral skills model in promoting adherence in CABG patients. J Health Psychol.

[35] Niiranen TJ, Leino K, Puukka P, Kantola I, Karanko H, Jula AM (2014). Lack of impact of a comprehensive intervention on hypertension in the primary care setting. Am J Hypertens.

